# Developing juvenile localized scleroderma (jLS) consensus treatment regimens for comparative effectiveness studies

**DOI:** 10.1186/1546-0096-10-S1-A68

**Published:** 2012-07-13

**Authors:** Suzanne C Li, Robert C Fuhlbrigge, Fatma Dedeoglu, Polly J Ferguson, Gloria C Higgins, Sandy D Hong, Heidi Jacobe, Andrew Lasky, Ronald M Laxer, Mimi C Morris, Elena Pope, C Egla Rabinovich, Kathryn S Torok

**Affiliations:** 1 Boston Childrens Hospital, Boston, MA, USA; 2 Brigham and Women's Hospital, Boston, MA, USA; 3 Childrens Mercy Hospital, Kansas City, MO, USA; 4 Duke University Medical Center, Durham, NC, USA; 5 Hackensack University Medical Center, Hackensack, NJ, USA; 6 Nationwide Childrens Hospital, Columbus, OH, USA; 7 Livingston, NJ, USA; 8 The Hospital for Sick Children, Toronto, ON, Canada; 9 U of Iowa Children's Hosp, Iowa City, IA, USA; 10 Univ of Pittsburgh Med Ctr, Pittsburgh, PA, USA; 11 UT Southwestern Medical Center, Dallas, TX, USA; 12 Stanford, CA, USA

## Purpose

LS can cause significant morbidity in the growing child, including joint contractures and facial and extremity hemiatrophy. Optimal therapy is not known, and few randomized clinical trials have been carried out. A prior survey of Childhood Arthritis and Rheumatology Research Alliance (CARRA) members identified methotrexate (MTX) and corticosteroids (CS) as the most commonly used medications to treat serious jLS. However, there was no consensus on dose, route of administration, or duration of treatment for these medications. Objective: To develop standardized consensus treatment regimens and disease assessments tools for jLS.

## Methods

A core group of pediatric rheumatologists based in CARRA was formed to evaluate and develop standardized jLS regimens and assessments. Two dermatologists who study jLS and a lay person were also recruited. Members reviewed literature on current treatments and assessments, and through surveys and Delphi processes, developed criteria to define different levels of disease severity, generated consensus regimens for jLS treatment, and agreed upon clinical parameters to evaluate disease activity and damage. Preliminary regimens and assessments were discussed with the CARRA membership, and modified based on feedback.

## Results

We have developed criteria to define high, moderate, and low disease severity, and standardized clinical activity and damage assessments for jLS. An atlas of lesion images was generated to facilitate scoring the level of several parameters (erythema, hyperpigmentation, hypopigmentation, dermal atrophy, and subcutaneous tissue loss). See table 1.

**Table 1:** Parameters are scored for each involved anatomic site

Clinical Activity Parameters:	Clinical Damage Parameters:
**Scoring levels**	**Scoring levels**

Erthema: 0 to 3	Dermal atrophy: None, shiny, visible vessel, cliff-drop
Violaceous color: None, lilac ring, viol. Center	Subcutaneous tissue atrophy: 0 to 3
Development in lesion size: Yes/no	Hyperpigmentation: 0 to 3
Change in lesion size: Smaller, stable, larger	Hypopigmentation: 0 to 3
Skin induration: lesion edge: 0 to 3	Skin thickness: lesion center: 0 to 3
Lesion warmth: Yes/no	
Distinct margin: None, erythematous, hyperpigmented margin	

Through use of the Delphi process, the jLS core group was able to generate consensus initial treatment regimens for MTX, MTX with oral CS, and MTX with intravenous CS, including a general tapering regimen for oral corticosteroids. Table 2.

**Table 2:** 

	Regimen		
	**MTX alone**	**MTX + CS**	**MTX + IV CS**

**MTX weekly dose**	1 mg/kg SQ (max 25 mg)	1 mg.kg SQ (max 25 mg)	1 mg.kg SQ (max 25 mg)

**Initial CS dose, duration**	None	2 mg/kg/day (max 60 mg) divided bid	30 mg/kg/dpse (max 1 gm)
		Duration: at least 2 weeks	Either: 3 consecutive daily doses per month
			OR 1 dose per week
			Duration: 3 months

**CS taper targets**	None	Down to:	None
		1 mg/kg/d (max 30 mg) by end of 2 months	
		0.5 mg/kg/d (max 15 mg) by end of 4 months	
		0.25 mg/kg/d (max 7.5 mg) by end of 6 months	
		Off CS by end of 48 weeks	

## Conclusion

There is a need for standardized jLS disease assessments and treatment regimens to be able to compare treatment efficacy. A CARRA subgroup has developed consensus assessments and treatment regimens for jLS. The efficacy of these regimens will be evaluated in future comparative effectiveness studies.

## Disclosure

Suzanne C. Li: Arthritis Foundation, 2, Friends of CARRA, 2, NIAMS-NIH, 2; Robert C. Fuhlbrigge: Arthritis Foundation, 2, Friends of CARRA, 2, NIAMS-NIH, 2; Fatma Dedeoglu: None; Polly J. Ferguson: None; Gloria C. Higgins: None; Sandy D. Hong: None; Heidi Jacobe: None; Andrew Lasky: None; Ronald M. Laxer: None; Mimi C. Morris: None; Elena Pope: None; C. Egla Rabinovich: None; Kathryn S. Torok: None; for the CARRAnet Investigators: None.

